# Selection Signatures from RAD-Seq Reveal Candidate Genomic Regions Potentially Underlying Immune and Adaptive Traits in Southwest Chinese Local Chickens

**DOI:** 10.3390/ani16182836

**Published:** 2026-09-09

**Authors:** Mengyu Wang, Mingyang Zou, Chenghao Zhou, Wei Han

**Affiliations:** 1 Jiangsu Institute of Poultry Science, Yangzhou 225125, China; w17826726279@163.com (M.W.);; 2 Shanghai Pudong New Area Animal Husbandry and Aquatic Products Technology Promotion Center, Shanghai 201299, China; 3 College of Animal Science and Technology, Yangzhou University, Yangzhou 225009, China; 4 Key Laboratory of Livestock and Poultry Resources (Poultry) Evaluation and Utilization, Ministry of Agriculture and Rural Affairs, Yangzhou 225125, China

**Keywords:** southwestern local chicken breeds, Fst and π ratio, domestication, selection signals, functional genes

## Abstract

Southwestern local chicken breeds in China have long been recognized for their strong adaptability and disease resistance, shaped by unique selective pressures during domestication. In this study, we combined Fst and π ratio analyses to detect selection signatures from RAD-seq data of four southwestern local breeds, Red Junglefowl, and 17 other Chinese local breeds. We identified 460 candidate genes under strong selection potentially associated with domestication, affecting behaviors, physiology, reproduction, immunity, and adaptability. Importantly, 34 genes showed strong and unique selection signals when comparing southwestern breeds with other local breeds. Among these, *CCR6*, *CD40LG*, and *CSF3R* represent candidate genes that have been previously associated with immune processes, while *MAPK10*, *FGFR4*, and *STAT3* represent candidate genes potentially related to disease resistance. Furthermore, *HTR1B, HSPB9*, and *RYR2* represent candidate genes potentially associated with environmental adaptability. Notably, *ALX1*, the only gene detected across all comparison groups, may reflect morphological adaptation related to feeding efficiency. Our findings provide valuable insights into the genetic basis of unique adaptations in southwestern local chicken breeds and highlight potential targets for breeding and conservation.

## Simple Summary

Local chicken breeds in Southwest China are highly valued for their remarkable ability to survive variable ecological environments and resist diseases, yet the genes responsible for these traits remain largely unknown. In this study, we analyzed DNA from four southwestern local chicken breeds and compared them with wild red junglefowl and other Chinese breeds. We discovered a number of genes that have been changed through domestication, many of which are candidate genes potentially involved in metabolism, behavior, growth, reproduction, and immunity. Thirty-four of these genes showed especially strong and unique changes in the southwestern breeds. Some of these genes have been reported to be associated with immune responses, while others are candidate genes potentially linked to environmental adaptation. One gene, called *ALX1*, which influences beak shape, was found in all comparisons, suggesting that changes in how chickens eat may have been important during domestication. These findings provide genetic evidence that may help explain why southwestern local chickens are so well adapted to their environments and provide breeders with valuable information to help preserve these unique genetic resources and improve chicken farming for the future.

## 1. Introduction

China has a long history of chicken domestication. In the oracle-bone inscriptions of the Yin and Shang dynasties, there were already hieroglyphs of chicken, which shows that domestic chicken was already a part of people’s lives at that time. Chicken provides important meat and egg resources for human beings, and it contains essential amino acids and proteins, which plays an indispensable role in maintaining the balance of human diet. Scientific research has revealed that the origin of domestic chickens is diverse, mainly dating back to South Asia, Southeast Asia and Southern China. The latest genomic study further indicates that the domestic chicken’s most likely direct ancestor is the *G. g. spadiceus*, which is distributed in southwest China, northern Thailand, and Myanmar [1]. This discovery highlights the significant role of China in the domestication and breed formation of chickens. Long-term processes of natural and artificial selection have given rise to a rich diversity of phenotypes and genotypes in local chicken breeds, providing valuable genetic resources for modern chicken breeding. Currently, many new breeds are developed based on the selection and improvement of these outstanding local breeds. However, the lack of genomic information on local chickens has been a major factor limiting modern breeding efforts. Deeply exploring and utilizing these genetic resources is of great significance for promoting the sustainable development and innovation of the poultry industry.

Against this backdrop, identifying selection signals associated with phenotypes plays a crucial role in explaining intra-species diversity. With the advancement of sequencing and gene analysis technologies, various statistical tests have been validated for their ability to detect signs of selection. Methods such as Fst based on population differentiation and π ratio based on heterozygosity have been widely used to identify genomic regions that may have been subject to selection. High-throughput sequencing technology has also facilitated the mining of functional genes, enabling a deeper understanding of the gene networks that control complex traits.

Chickens in the southwestern region, due to geographical isolation, as well as differences in feeding management and environmental conditions across various regions, have evolved distinct traits related to immune adaptability and resistance to coarse feed. In particular, small-bodied local breeds such as the Tibetan chicken, Chahua chicken, Daweishan Mini chicken, and middle-bodied Piao chicken, which are close to the Red Junglefowl, are important subjects for multi-omics research. These local breeds not only possess unique genetic features but also serve as valuable resources in the study of chicken genetic diversity due to their adaptation to diverse environmental conditions. The Tibetan chicken, in particular, has attracted considerable attention for its remarkable adaptability to high-altitude hypoxic environments [2,3]. Chahua chicken, Daweishan Mini chicken, and Piao chicken are also gradually gaining the attention of researchers due to their unique genetic characteristics.

This study employed Restriction-site Associated DNA sequencing (RAD-seq) to genotype and identify SNPs in 21 local chicken breeds in China. Using a combined approach of Fst and π ratio, we detected selection signals in local chicken breeds from the southwestern region, and screened for functional genes that have been under strong selection during long-term domestication and selection processes. This work aims to enhance our understanding of the evolutionary and genetic foundations of these southwestern local chicken breeds and to provide a scientific basis from a genetic perspective for their use as valuable breeding materials for local chicken breeds in the region.

## 2. Materials and Methods

### 2.1. Sample and Source

Considering the phenotypic characteristics and geographical distribution, blood samples were collected from 21 local chicken breeds, among which 4 are from the southwestern region, including Tibetan Chicken, Piao Chicken, Chahua Chicken, and Daweishan Mini Chicken; the others include Langya Chicken, Xiaoshan Chicken, Luyuan Chicken, Beijing You Chicken, Dongxiang Blue-shell Egg Chicken, Wenchang Chicken, Bian Chicken, Langshan Chicken, Baier Yellow Chicken, Xianju Chicken, Anyi Grey Chicken, Gushi Chicken, Dagu Chicken, Huiyang Bearded Chicken, Wenshang Luhua Chicken, Jinhu Black-Bone Chicken, and Tianjin Monkey Chicken.

Samples from pure breed populations were collected from the National Local Chicken Gene Bank in Jiangsu, China, with the exception of the Tianjin Monkey Chicken, which was sampled from the Institute of Animal Husbandry and Veterinary Medicine of Tianjin Academy of Agricultural Sciences. Genomic DNA was extracted from the blood samples using the standard phe-nol-chloroform method. The quality and concentration of the DNA were assessed using a fluorescence spectrophotometer and gel electrophoresis. The sequences for Red Jungle-fowl were sourced from the NCBI database.

### 2.2. Sequencing and Quality Control

The ddRAD library construction method was used to create pair-end libraries (300~500 bp), and double enzyme digestion RAD-seq was performed using EcoRI (G^AATTC) and NlaIII (Hin1IIXATG^). The prepared libraries were sequenced to identify genome-wide SNP markers. Sequencing data were aligned to the chicken reference genome (GRCg6a) using BWA MEM 0.7.15. Quality control criteria that included (1) removing markers with Minor Allele Frequency (MAF) less than 0.05; (2) removing markers with a genotype call rate below 70% across all samples; and (3) removing markers with a genotype call rate below 90% within any individual breed.

### 2.3. Data Analysis

Based on the geographical distribution and historical formation of the breeds, the Neighbor-Joining (NJ) method was used to construct a genetic phylogenetic tree (Plink v1.9 and the bionj function of R v4.3.1). To determine genome-wide selective sweeps related to domestication and selection, the fixation index (Fst) values and π ratio were calculated for the defined group pairs (vcftools v0.1.16). The Fst values were plotted in 100 kb genomic bins with a 10 kb step. Then, the nucleotide diversity (pi) was estimated for the same bins. For each contrast, π ratio was calculated as π_reference/π_XNC, where reference denotes RJF or the corresponding domestic group (A–E). A positive log2 (π ratio) value indicates reduced nucleotide diversity in the XNC group relative to RJF, consistent with the expected signature of positive selection. Windows that simultaneously possess the top 5% of Fst and log2 (π ratio) values are considered to be putative selective sweeps under the specified outliers. Windows with positive signals are annotated using Bedtools to obtain candidate genes. These genes are then uploaded to online tools (http://bioinfo.org/kobas/ (accessed on 3 September 2026))for KEGG enrichment analysis to further explore the functions of the candidate genes.

## 3. Results

### 3.1. Phylogenetic Analysis of Local Chicken Breeds in Southwest China

The samples selected for the study took into account the characteristic characteristics and environmental conditions. After RAD-seq and quality control, 365K SNP sites were obtained.

**Figure 1:**
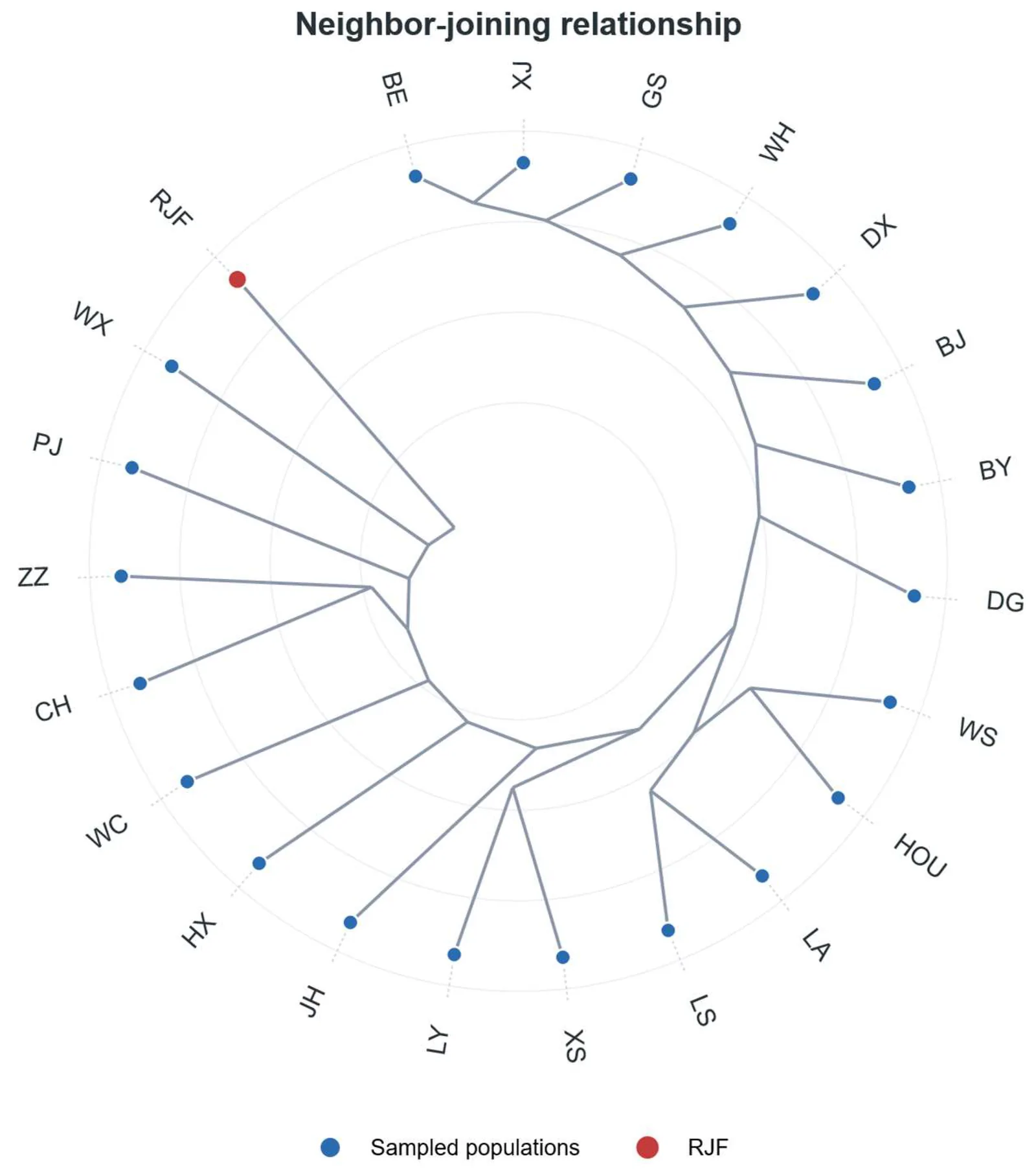
Phylogenetic tree based on NJ.

Based on SNP markers for phylogenetic analysis (Figure 1). The Red Junglefowl (RJF) is considered the ancestor of local chicken breeds and serves as the root of the evolutionary tree. Local chicken breeds are divided into six major groups according to their branch distribution (Table 1). The first group includes four breeds from the southwestern region: Daweishan Mini Chicken, Tibetan Chicken, Chahua Chicken, and Piao Chicken (XNC); the second group consists of Wenchang Chicken and Huiyang Bearded Chicken (Group A); the third group includes Tianjin Monkey Chicken, Wenshang Luhua Chicken, Langya Chicken, and Langshan Chicken (Group B); the fourth group includes Bian Chicken, Dagu Chicken, Beijing You Chicken, Anyi Grey Chicken, Dongxiang Blue-shell Egg Chicken, Gushi Chicken, Xianju Chicken, and Baier Yellow Chicken (Group C); the fifth category includes Luyuan Chicken and Xiaoshan Chicken (Group D); and the sixth group is the relatively independent Jinhu Black-Bone Chicken (Group E).

**Figure A1:**
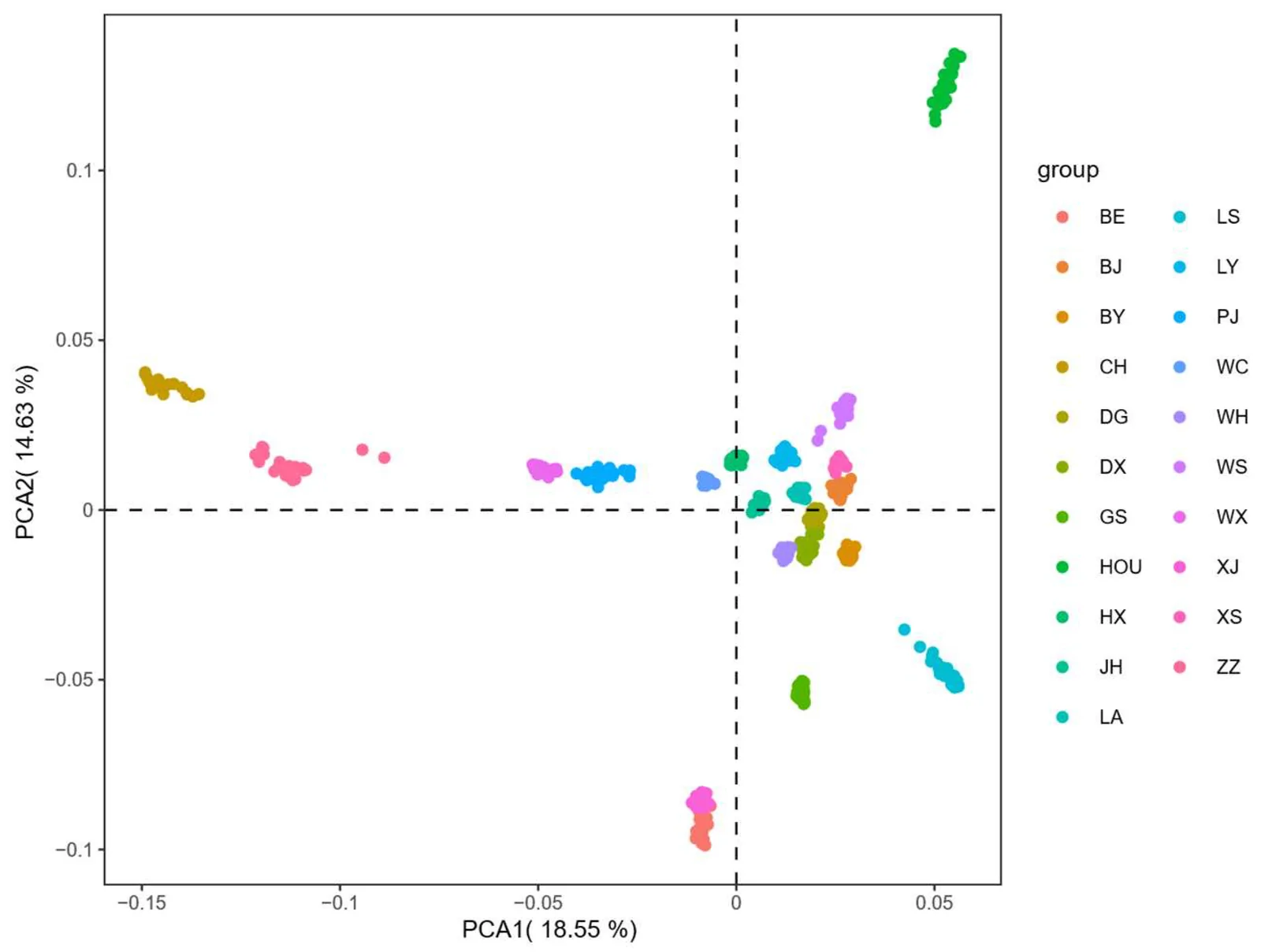
Principal component analysis (PCA) plot of 21 Chinese local chicken breeds based on RAD-seq SNP data.

To further validate the grouping of the four southwestern breeds as a single analytical population, we performed PCA based on RAD-seq SNP data from all 21 breeds. The PCA plot (Figure A1) showed that individuals from the four XNC breeds (Tibetan, Piao, Chahua, and Daweishan Mini chickens) clustered together in the same region of the plot, with genetic distances among them being smaller than those separating them from other breed groups, supporting their treatment as a unified population in subsequent analyses.

### 3.2. Selection Signals in Comparison to the Red Junglefowl

We used Fst and π ratio to identify genomic regions in Southwest local chicken breeds that may have been targets of positive selection. Merge the SNPs within the XNC group, calculate the Fst value of the XNC vs. RJF group, and calculate πRJF/πXN.

**Figure 2:**
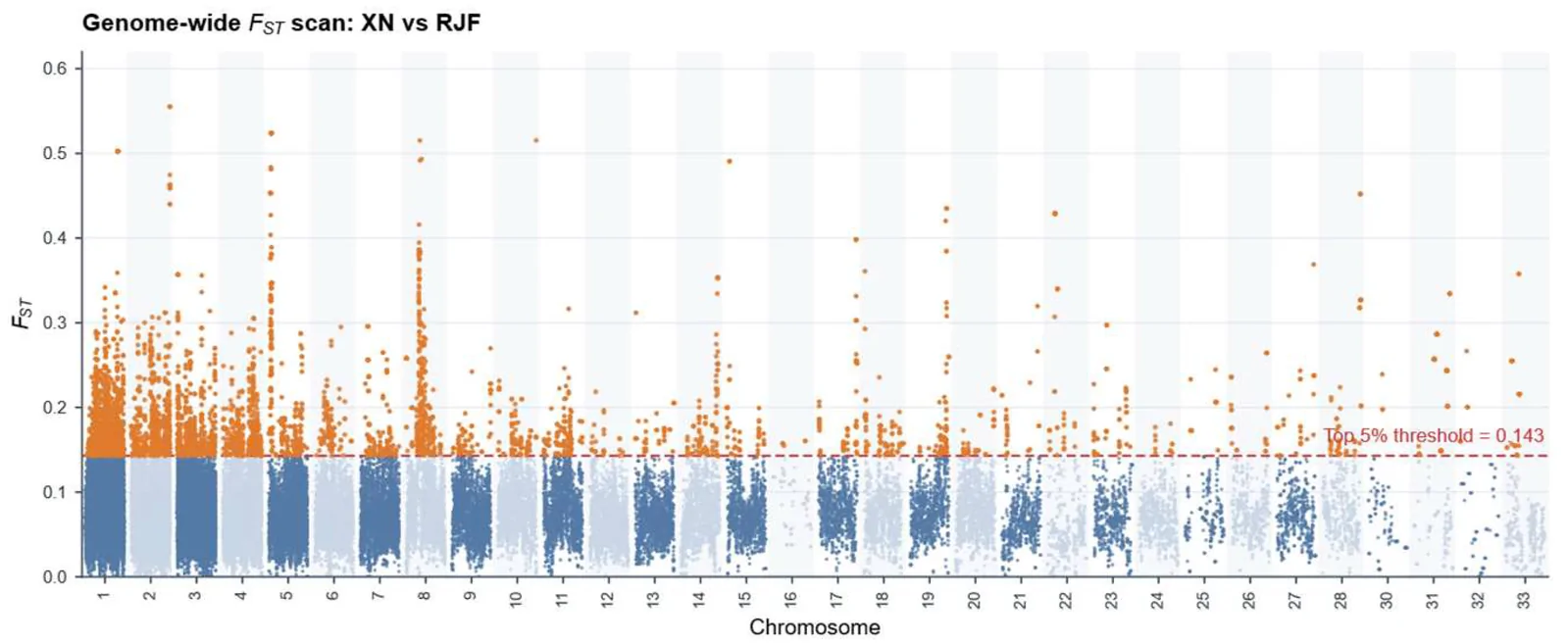
Distribution of Fst calculated using 100 kb windows with a 10 kb sliding window showing the genes identified and selected in the XNC (WX, ZZ, CH, PJ) group’s genome as compared with RJF. Labeled genes have extreme Fst values (top 5% level) in the XNC candidate genes.

**Figure 3:**
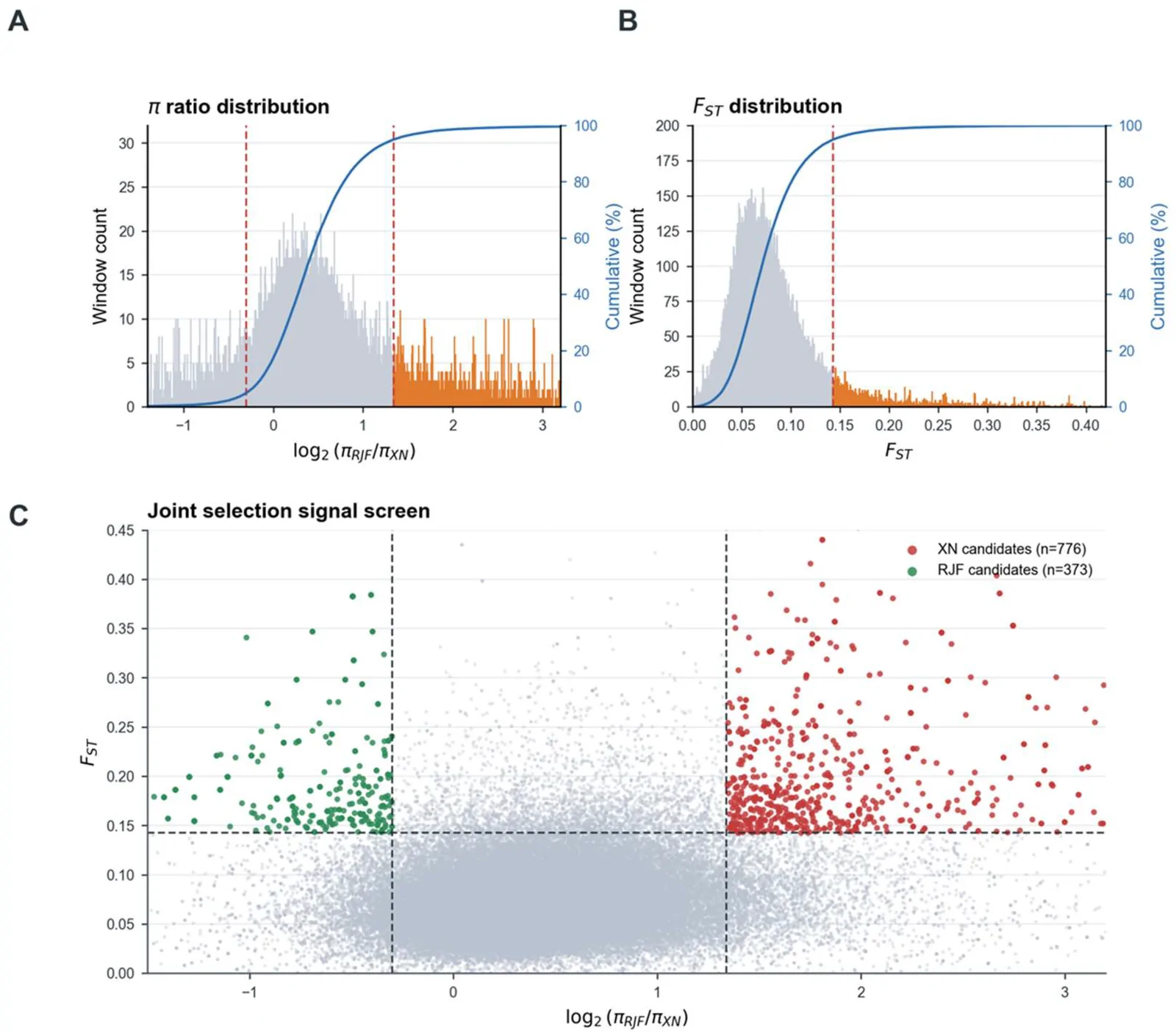
Frequency distributions of π ratio (log2 transformed) (**A**) and Fst (**B**). In panels (**A**,**B**), orange marks indicate windows that exceed both candidate thresholds and contain annotated genes. (**C**) Selective sweep mapping between XNC and RJF. The top 5% outlier windows are marked in green and red, both indicating elevated selection signals.

**Figure 4:**
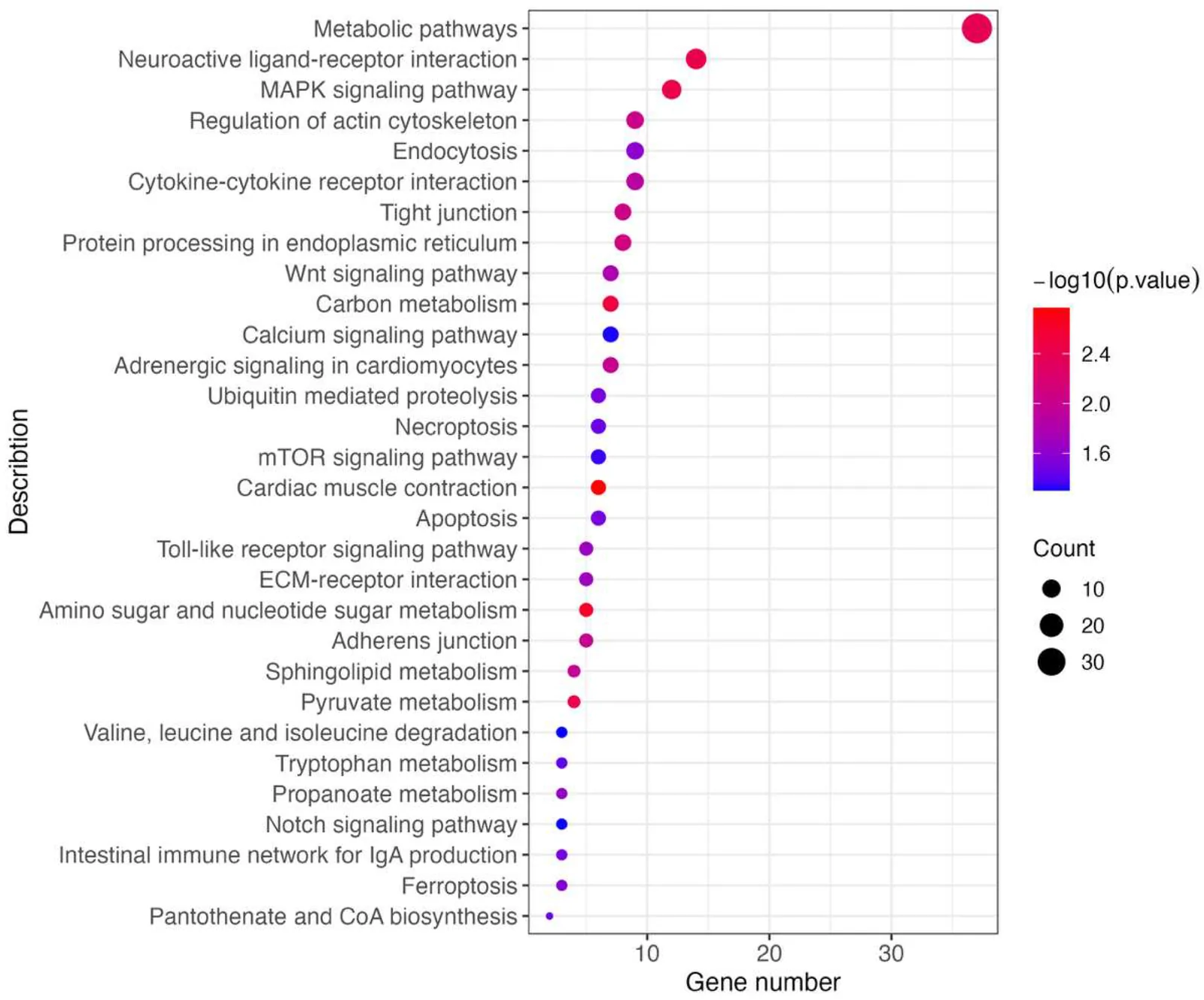
The KEGG pathway of the 460 gene under selections in XNC vs. RJF (*p* < 0.05).

Fst reflects the population differentiation of Southwest local chicken breeds (Figure 2), with regions above the top 5% level (Fst > 0.143) showing elevated population differentiation signals. Based on selection signals, we identified 772 windows as candidate regions using thresholds of Fst > 0.143 and log2 (π ratio) > 1.88. And a total of 460 genes were annotated within these regions (Figure 3). These genes were considered candidate genes located in genomic regions showing selection signatures. The 460 candidate genes were collectively enriched in 30 KEGG pathways (*p* < 0.05), which are associated with physiological structures, metabolism, behavior, reproduction, and immunity (Figure 4).

**Figure 5:**
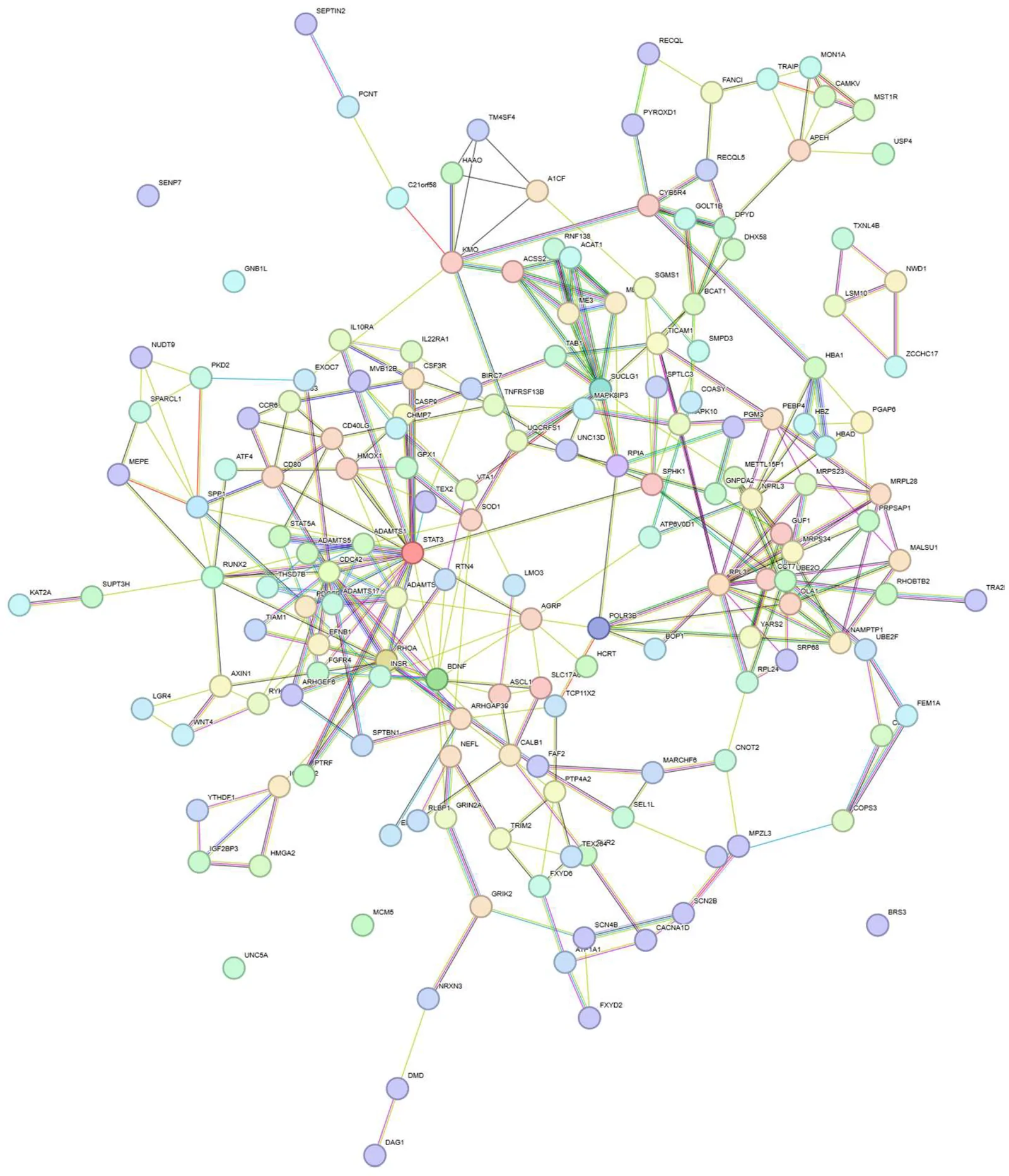
Protein-protein interaction network of the 167 core genes based on the STRING database.

**Figure 6:**
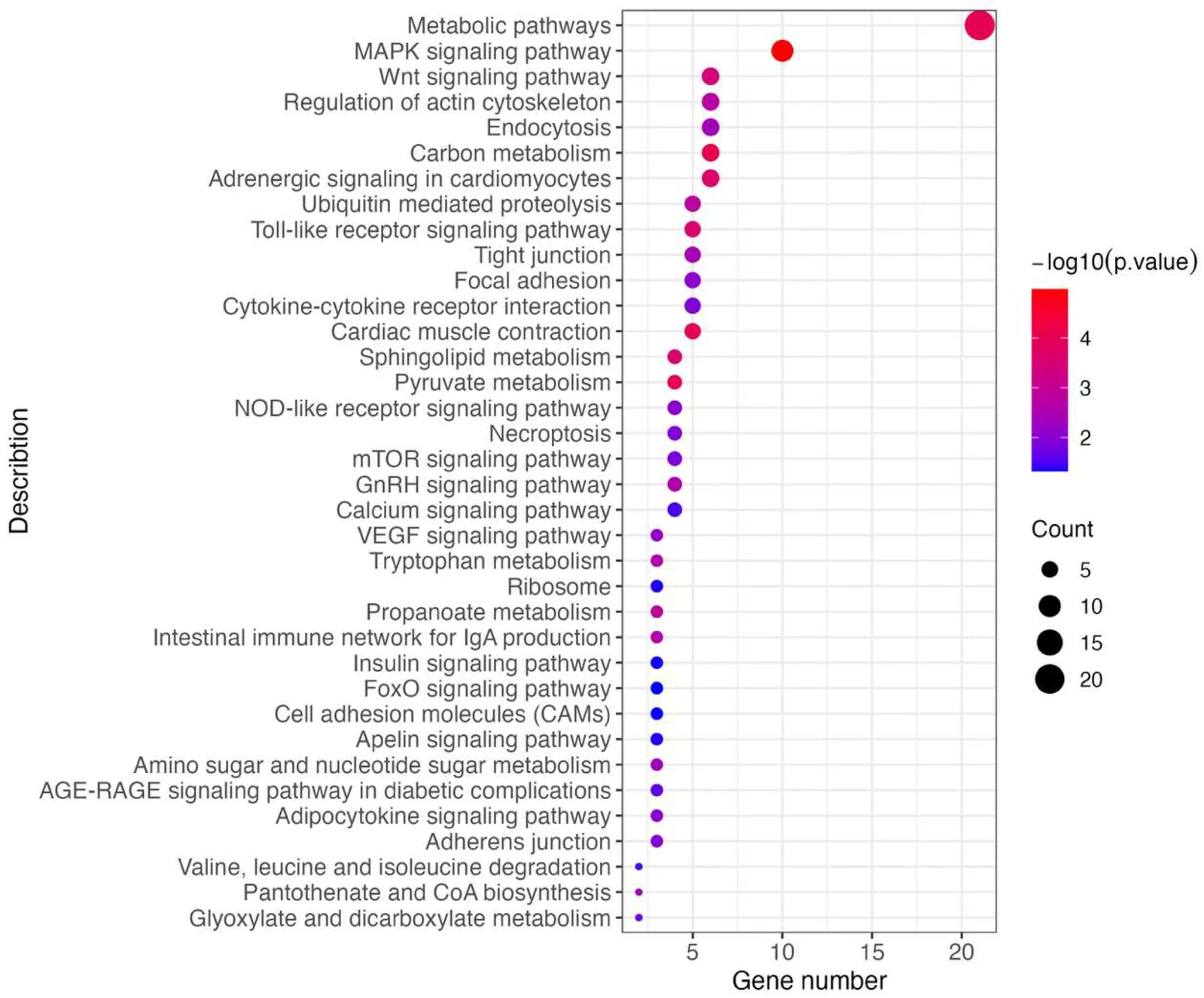
The KEGG pathway of the 167 core gene under selection in XNC vs. RJF (*p* < 0.05).

A protein-protein interaction (PPI) network for the 460 functional genes was constructed using the STRING database (https://string-db.org/), which subsequently identified 167 core genes (Figure 5). The results of the KEGG pathway enrichment analysis are presented in Figure 6. The 167 genes were mapped to 36 KEGG pathways (*p* < 0.05).

### 3.3. Formatting of Mathematical Components

**Figure 7:**
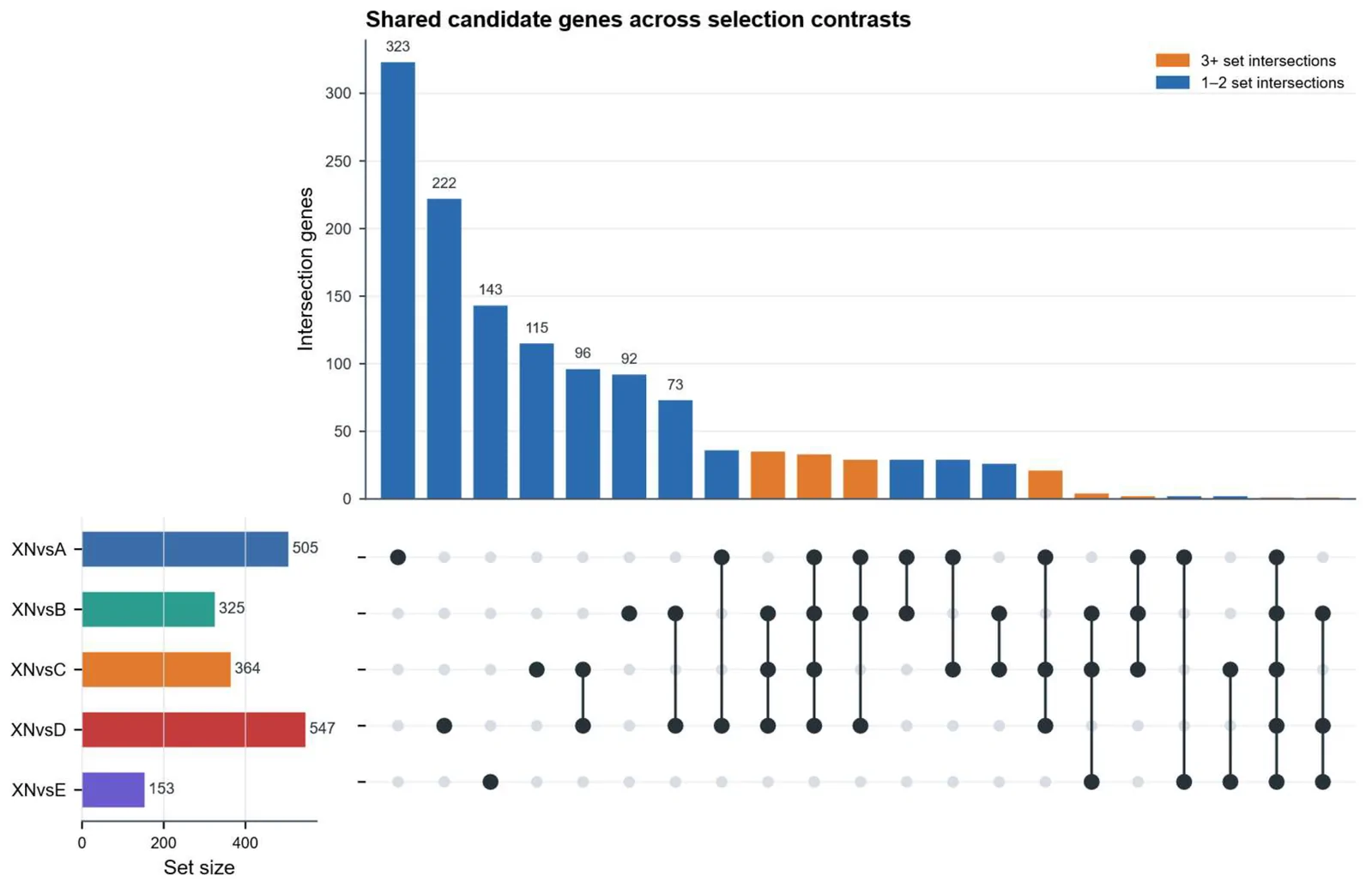
Intersection of candidate genes in different groups of XNC.

Local chicken breeds from different geographical regions exhibit distinct genetic variants and genomic features, reflecting a combination of environmental adaptation, founder effects, genetic drift, and artificial selection during domestication. By conducting combined selection signal detection using Fst and π ratio between XNC and groups A, B, C, D, and E, with the top 5% considered as candidate genes, we identified 505 (XNC vs. A), 325 (XNC vs. B), 364 (XNC vs. C), 547 (XNC vs. D), and 154 (XNC vs. E) candidate genes, respectively. Figure 7 displays the intersections of candidate genes from the Southwestern local chicken populations across different comparison groups. Applying the stringent criterion that a selected gene must be identified in at least four comparative groups, we screened 34 candidate genes showing strong selection signals for these populations (Table 2). Among these, only a single gene, *ALX1*, was detected across all comparison groups. The remaining 33 genes were predominantly concentrated in the comparisons between the XNC group and groups A, B, C, and D.

## 4. Discussion

In this study, we employed RAD-seq to genotype 21 Chinese local chicken breeds and applied a combined Fst and π ratio approach to detect selection signatures associated with domestication and local adaptation. By comparing four southwestern breeds with Red Junglefowl and other indigenous populations, we identified 460 candidate genes that may have been targets of selection during domestication and 34 putatively selected genes showing consistent signals across multiple comparative groups. These candidate genes are enriched in pathways related to metabolism, behavior, growth, reproduction, immunity, and environmental adaptation, providing valuable genomic insights into the adaptive evolution of southwestern local chickens.

Nevertheless, several limitations should be considered when interpreting these findings. The candidate genes reported here were annotated from genomic windows showing selection signatures, and as RAD-seq captures SNP markers distributed across the genome—many in non-coding regions and potentially in linkage disequilibrium with causal variants—functional assays and fine-mapping are required to confirm their roles. In addition, sample sizes were uneven across breeds (RJF: *n* = 10; Huiyang Bearded: *n* = 20; others: *n* = 30). While the top 5% outlier approach based on relative rankings rather than absolute values partially mitigates this concern, results should still be interpreted with caution. Additionally, pooling the four southwestern breeds into a single XNC group may dilute breed-specific signals.

With these considerations in mind, we next discuss the candidate genes and pathways identified in this study.

### 4.1. Genes and Pathways Associated with Domestication

Metabolism is a complex process. During the domestication from wild chickens to local chicken breeds, changes in food types and feeding habits can lead to metabolic adaptations in poultry. In KEGG enrichment analysis, most pathways are associated with metabolism. For instance, the Metabolic pathways encompass a broad range of metabolic reactions, potentially involved in the activation and transformation of energy substrates in chickens. The Amino sugar and nucleotide sugar metabolism pathway is linked to the digestion and absorption of starch and proteins, which is crucial for adapting to high-energy, high-protein, and high-starch diets. Additionally, the Carbon metabolism pathway plays a key role in energy metabolism and is significantly enriched in chicken liver, directly related to the regulation of carbohydrate metabolism efficiency from dietary sources [34]. The enrichment of these metabolic pathways suggests that genes involved in carbohydrate and energy metabolism may have been targets of selection during the domestication of southwestern local chicken breeds, potentially reflecting adaptation to diverse feed resources and feeding practices.

Affinity of animals toward humans is a primary prerequisite for domestication. The development of the nervous system has been demonstrated to play a crucial role in the domestication process of dogs [35]. In this study, several behavior-related KEGG pathways were significantly enriched (*p* < 0.05). For example, Neuroactive ligand-receptor interaction pathway is associated with the regulation of behavioral patterns and stress responses [36], while Adrenergic signaling in cardiomyocytes pathway may indirectly influence behavioral adaptation in chickens by modulating cardiac contraction and stress reactivity [37]. After domestication, chickens exhibited reduced vigilance in predator avoidance and startle responses, becoming more docile. This study identified several genes under strong selection pressure related to behavioral adaptation, such as *AXIN1* and *IL1RAPL1*. Fear responses are linked to differential gene expression in the brain, and *AXIN1* may serve as a molecular regulator of fear responses in poultry [38]. Additionally, *IL1RAPL1* has been implicated in neural development in Luxi gamecocks [39]. Knockout of *IL1RAPL1* in mice leads to reduced spine density, affecting not only learning but also behavioral flexibility, locomotor activity, and anxiety-like behaviors [40]. While this function has not been directly validated in chickens, the observed selection signal in *IL1RAPL1* suggests a potentially conserved role in neural development that warrants further investigation in poultry.

Compared to red junglefowl, domesticated chickens exhibit significantly reduced flight capacity, accompanied by various physiological and structural adaptations resulting from domestication. Red junglefowl possess a smooth sternal surface with a more curved and serrated keel (carina sterni), along with a higher proportion of pectoralis superficialis and profundus muscles (relative to body weight), which facilitate flight. In contrast, domestication has led to a reduction in keel curvature, decreased serration, the appearance of pneumatic foramina and depressions on the sternal surface, and a lower ratio of pectoral muscles [41,42]. These modifications collectively diminish flight capability in domestic chickens. In this study, the Regulation of actin cytoskeleton pathway was significantly enriched. This pathway plays a pivotal role in cellular morphology maintenance and motility [43] and may also regulate sternal development during chicken domestication. Among the candidate genes showing selection signals identified, *FGFR1* and *BMP3* are likely involved in adaptive skeletal modifications in local chicken breeds. Specifically, *FGFR1* has been shown to modulate bone mass via the Wnt/β-catenin signaling pathway in mouse osteocytes [44], suggesting a potential role in skeletal development that may also apply to chickens. Meanwhile, *BMP3* expression in the chick perichondrium regulates chondrocyte proliferation [45], supporting its candidacy as a gene involved in skeletal modification during chicken domestication.

The significant enrichment of KEGG pathways such as MAPK signaling pathway, Notch signaling pathway, TGF-beta signaling pathway, etc. showed the changes in growth and development of chickens during domestication. Compared to red junglefowl, domesticated chickens typically exhibit faster muscle growth, increased meat yield, and distinct muscle fiber characteristics. In this study, several genes under strong selection pressure were identified as being associated with muscle development and growth, such as *IGFBP2*, *ACAT1*, *IGFBP3*, *VGLL2*, *NELL1*, *SMPD3*, *GNB1L*, *PDGFB, HK3*, and *MEF2A*. Notably, *IGFBP2*, an introgressed allele from gray junglefowl [46], functions as an inhibitor of skeletal muscle development in domestic chickens. *ACAT1* has been reported to negatively regulate growth rate in chickens [47], while *IGFBP3* [48], *VGLL2* [49], *NELL1* [50] and *SMPD3* [50] have been suggested to promote muscle development. Additionally, *GNB1L* [51], *PDGFB* [52], *HK3* [53], and *MEF2A* [54] have been implicated in growth regulation in chickens. Furthermore, *PRKAG3* contains a SNP that significantly influences muscle fiber density [55], highlighting the genetic complexity underlying diverse muscle phenotypes during domestication.

Domestication is a human-driven process in which preferences for meat quality traits, including fat deposition, have been shaped through artificial selection. In this study, several enriched KEGG pathways were identified as classical pathways associated with fat deposition, including Fatty acid degradation, Insulin signaling pathway, and Adipocytokine signaling pathway. Concurrently, we identified several candidate genes potentially responsible for adaptive changes in fat deposition, such as *ITGB2*, *MGAT3, SPHK1*, *BCAT1*, and *COL6A1*. The *ITGB2* gene is strongly associated with the differentiation of abdominal adipocytes [56]. *MGAT3* has been reported to regulate the adipogenic differentiation of chicken abdominal pre-adipocytes [57]. *SPHK1* is pivotal in fatty acid deposition [58]. *BCAT1* is implicated in the regulation of muscle lipid synthesis metabolism [59]. Furthermore, elevated methylation in the promoter region of *COL6A1* could account for the notable variations in chicken meat quality observed across different egg-laying periods [60].

Comparative analysis with the Red junglefowl revealed enrichment of reproduction-related pathways including GnRH signaling pathway, Steroid hormone biosynthesis, Progesterone-mediated oocyte maturation, and Wnt signaling pathway. Several candidate genes under strong selection signals were identified, including *WNT4*, *PDE1C*, *NEDD4*, *FANCI*, *HSD17B1*, *KIF18*, *SPP1*, *SLCO1A2* and *MITF*. The Wnt signaling pathway plays crucial roles in ovarian and follicular development, where *WNT4* participates in chicken follicle selection and development by upregulating follicle-stimulating hormone receptor expression [61]. *PDE1C* is expressed in oviduct ciliated and secretory cells, regulating oviductal fluid flow [62] and potentially affecting sperm storage duration [63]. Both *NEDD4* [64] and *FANCI* [65,66] have been reported to be associated with reproductive function in mammalian studies, suggesting potential roles that warrant investigation in chickens. *HSD17B1* is essential for regulating estradiol levels [67], follicular maturation, and egg production [68]. Furthermore, *KIF18* is involved in reproductive processes [1] and associated with laying performance in Tibetan chickens [50]. *SPP1* influences eggshell quality [26], while *SLCO1A2* [69] and *MITF* [70] genes participate in eggshell color regulation. 

Collectively, these identified pathways and genes provide valuable insights into the adaptive changes during the domestication of chickens, represented by the Southwestern local breeds, particularly in metabolism, behavior, growth, skeletal structure, reproduction, and fat deposition.

### 4.2. Genes and Pathways Associated with Immunity

Immunity encompasses both general disease resistance and pathogen-specific immunity, whereas adaptability represents a broader concept involving an organism’s integrated response to diverse environmental stresses, including both specific and nonspecific defense mechanisms against pathogens. This generalized disease resistance and adaptive capacity are critical for survival in dynamic environments. In poultry production, immune function and adaptability directly influence survival rates and growth performance, thereby determining farming profitability. Consequently, identifying key genes related to immunity and environmental adaptation in poultry breeds that thrive in specialized ecological conditions is critically important for advancing genetic improvement programs in the poultry industry.

Human breeding practices have significantly influenced the immune system of chickens. Over the course of domestication, chickens have been continuously exposed to diverse pathogens in human-managed environments. This process enhances disease resistance and leads to diversification of immune system functions in chickens. KEGG enrichment analysis identified multiple pathways associated with general disease resistance in Southwest local chicken breeds, including Cytokine-cytokine receptor interaction, Toll-like receptor signaling pathway, Endocytosis, Apoptosis, Intestinal immune network for IgA production, Necroptosis, Neomycin, kanamycin and gentamicin biosynthesis, NOD-like receptor signaling pathway, Phagosome, RIG-I-like receptor signaling pathway and C-type lectin receptor signaling pathway. A number of genes are involved in diverse immune regulatory mechanisms, such as *STAT4*, *CCR6*, *CD40LG*, *TNFRSF13B*, *CSF3R*, *FPOLG*, *DAD1*, *TICAM1* and *IFNL3*. *STAT4* participates in TH1 cell differentiation and regulates immune cell development and function through cytokine interactions [71]. *CCR6* has been reported to control the transport of dendritic cells [72], while *CD40LG* enhances T cell immune responses [73]. *TNFRSF13B* is significantly enriched in the intestinal immune network of the IgA production signaling pathway [74]. The *CSF3R* gene is linked to macrophage function and innate immunity [75]. *CD80* is expressed in chicken splenic lymphocytes and participates in avian mucosal immunity [76]. *FPOLG* is essential for in mitochondria DNA maintenance and influences immune responses to mitochondrial diseases [77]. Additionally, *DAD1* [78], *TICAM1* [79,80] and *IFNL3* [81] are key innate immunity factors, with the function of *IFNL3* also being reported in Indian Sahiwal cattle [82].

Several pathways associated with host-pathogen interactions were significantly enriched, including Herpes simplex virus 1 infection, Influenza A, and Salmonella infection pathways. Additionally, we identified multiple candidate genes showing selection signals associated with pathogen resistance. *FGFR4* has been implicated in the molecular regulation of host responses to Avian Leukosis Virus Subgroup J [83]. The expression of *MAPK10* in the cecal tissue of chickens correlates with Eimeria infection [84], and *STAT1* [85] has been reported to be associated with Salmonella resistance in ducks [86]. *ATF4* and *CDC42* are involved in Newcastle disease virus infection, particularly in B cell differentiation [87,88]. *TAB1* activation in response to infectious bronchitis virus infection enhances pro-inflammatory factors IL-1β and TNF-α through the NF-κB signaling pathway [89]. *XPO1* mediates the export of viral proteins related to respiratory syncytial and influenza viruses [90] and influences IBV replication through protein interactions [91].

Through selective sweep analysis of Southwest local chicken breeds and other indigenous chicken populations, we identified 34 candidate genes showing positive selection signals with important biological functions. Among these, *NOTCH2* has been demonstrated to regulate B-cell development in mammalian models [16,92] and plays a critical role in immune responses. *SRSF1* is involved in the late development and maturation of thymic T cells [18], thus supporting normal immune system function. The *FKBP4* gene encodes *FKBP52* protein, which negatively regulates glucocorticoid production [13]. *FOXP4* promotes human tumorigenesis and progression by destabilizing protein structures and altering protein-phase interactions [14]. *ITFG2* modulates B-cell differentiation while negatively regulating mTORC1 signaling transduction; it also protects the heart from ischemic injury by regulating mitochondrial function through interaction with *ATP5B* [15]. *PLPP6* regulates inflammatory responses in humans, being expressed in neutrophils and lung dendritic cells. It modulates cellular cholesterol content and macrophage pinocytosis, playing a key regulatory role in the pathological mechanisms of allergen-induced pulmonary inflammation in mice [17].

Collectively, these candidate genes offer insights into the genetic basis of immunity in southwestern local chickens. While some have been directly studied in chickens and support their involvement in avian immunity, others are inferred from mammalian models and await functional validation. Nonetheless, the selection signals observed in these candidate genes highlight their potential importance in the immune adaptation of southwestern local chicken breeds and warrant further investigation.

### 4.3. Genes Associated with Environmental Adaptation

The complex and variable geographical environment and climatic conditions in Southwest China have driven the evolution of adaptive mechanisms in local chicken breeds. Our study identified *HTR1B*, *LGR4*, *HCRT*, *RYR2*, *INSR*, and *HSPB9* as potential key genes involved in adaptation to temperature fluctuations. *HTR1B* [93,94], *LGR4* [95], and *HSPB9* [96,97] have been implicated in thermal adaptation across various species, including freshwater leeches, chickens, goats, and cattle. The *HSPB9* may confer cellular protection against heat stress through elevated expression [98]. In contrast, *HCRT* [99], *INSR* [100], and *RYR2* [101,102] exhibit significant expression changes in cold environments, potentially linking their expression variations to metabolic regulation and energy balance, which are crucial for adapting to low temperatures. Additionally, *SLC9A5* has been shown to mediate adaptation to extreme alkaline environments in Amur ide fish [103]. These findings suggest that these candidate genes may have played a role in the environmental adaptation of local chickens. However, it should be noted that some of these genes, including *HSPB9* and *HTR1B*, are widely expressed genes that have been implicated in stress responses across multiple species. Their detection in our selection scan does not necessarily establish them as the causal drivers of environmental adaptation, as they may be linked to unsequenced functional variants through linkage disequilibrium. Functional validation is required to confirm their specific roles in chicken adaptation.

### 4.4. ALX1 and Morphological Adaptation

Our study identified *ALX1* as a particularly strong selection signal in Southwest local chicken breeds. Research has demonstrated that *ALX1* is closely associated with beak morphology diversification in passerine birds, enabling expanded food resource utilization [32]. In their natural habitat, red junglefowl rely on slender, pointed beaks for multiple survival functions: capturing insects from narrow crevices, foraging efficiency, preening feathers for courtship and parasite removal, as well as defensive combat. While Southwest local chickens, having adapted to semi-wild environments, show no significant selection pressure on *ALX1* compared to red junglefowl, they exhibit strong selection signals relative to other domesticated breeds. This suggests selection on *ALX1* may be linked to adaptations in feeding ecology and behavior during domestication. Previous studies have documented substantial morphological divergence between domesticated chickens and their wild ancestor, the red junglefowl, including disproportionate beak growth during development [104]. It has been speculated that such morphological changes may reflect adaptations in feeding ecology, though this hypothesis has not been systematically tested across breeds. Direct morphometric evidence linking *ALX1* variation to beak morphology in chickens is lacking in the present study, and targeted functional assays are needed to validate its role. Notably, *ALX1* mutations are known to cause frontonasal dysplasia in humans [31,105] and play crucial roles in vertebrate neural crest cell differentiation, highlighting its evolutionary conservation across vertebrates. Given this conserved role, further investigation into *ALX1* regulatory mechanisms in poultry not only advances our understanding of avian evolutionary adaptations but also provide insights relevant to human developmental biology.

## 5. Conclusions

This study identified a suite of candidate genes showing strong selective signatures in southwestern Chinese local chickens, providing genomic evidence for their adaptive evolution. Functional enrichment analysis highlighted key pathways related to metabolism, behavior, growth, and immunity, with candidate genes potentially underpinning these traits. Notably, genes associated with beak morphology and immune adaptation were among the putatively selected candidates. Collectively, these findings deepen our understanding of the genetic architecture driving local adaptability and disease resistance in these indigenous populations. Moreover, the identified genomic regions and candidate genes represent valuable resources for marker-assisted selection and conservation strategies, potentially facilitating the sustainable utilization of southwestern chicken germplasm resources in future breeding programs.

## Figures and Tables

**Table 1:** Breed and Grouping Information.

Breeds	Abbreviation	Quantity	Origin	Source	Group
Huiyang Bearded Chicken	HX	20	Guangdong, China	α	A
Wenchang Chicken	WC	30	Hainan, China	α	A
Langshan Chicken	LS	30	Jiangsu, China	α	B
Langya Chicken	LA	30	Shandong, China	α	B
Tianjin Monkey Chicken	HOU	30	Tianjin, China	β	B
Wenshang Barred Chicken	WS	30	Shandong, China	α	B
Anyi Grey Chicken	WH	30	Jiangxi, China	α	C
Baier Yellow Chicken	BE	30	Jiangxi, China	α	C
Beijing You Chicken	BY	30	Beijing, China	α	C
Bian Chicken	BJ	30	Inner Mongolia, China	α	C
Dagu Chicken	DG	30	Liaoning, China	α	C
Dongxiang Blue-Shelled Egg Chicken	DX	30	Jiangxi, China	α	C
Gushi Chicken	GS	30	Henan, China	α	C
Xianju Chicken	XJ	30	Zhejiang, China	α	C
Luyuan Chicken	LY	30	Jiangsu, China	α	D
Xiaoshan Chicken	XS	30	Zhejiang, China	α	D
Jinhu Black-Bone Chicken	JH	30	Fujian, China	α	E
Red Junglefowl	RJF	10	South Asia, Southeast Asia	γ	RJF
Chahua Chicken	CH	30	Yunnan, China	α	XNC
Daweishan Mini Chicken	WX	30	Yunnan, China	α	XNC
Piao Chicken	PJ	30	Yunnan, China	α	XNC
Tibetan Chicken	ZZ	30	Tibet, China	α	XNC

Note: α represents the National Local Chicken Breed Gene Bank (Jiangsu) Protected population, β represents the Institute of Animal Husbandry and Veterinary Medicine of Tianjin Academy of Agricultural Sciences and γ represents NCBI database: PRJEB42301.

**Table 2:** 34 Functional genes with elevated selection signal from XNC.

Symbol	Annotation	Class
*DUT*	Nucleotide metabolism (https://www.genecards.org/)	Metabolism
*KCNA5*	Potassium channel (https://www.genecards.org/)	Metabolism
*REG4*	The jejunum morphology of broilers [4]	Metabolism
*SLC12A1*	Kidney-specific transport proteins (https://www.genecards.org/)	Metabolism
*CUEDC1*	Follicular development [5]	Reproduction
*FAM78B*	The reproductive traits of sheep [6]	Reproduction
*FBN1*	Improve protein quality [7]	Reproduction
*KDM7A*	Egg quality traits [8]	Reproduction
*MRPS23*	laying rate [9]	Reproduction
*NTF3*	Testicular development [10]	Reproduction
*TEAD4*	Primordial follicular development [11]	Reproduction
*HMGCS2*	sperm motility [12]	Reproduction
*FKBP4*	Negative regulatory glucocorticoids [13]	Immunity
*FOXP4*	Human tumorigenesis [14]	Immunity
*ITFG2*	Immunomodulatory cell proteins [15]	Immunity
*NOTCH2*	Regulate B cells [16]	Immunity
*PLPP6*	Inflammatory regulation [17]	Immunity
*SRSF1*	Thymus T cell development and maturation [18]	Immunity
*TULP3*	Cause diseases [19]	Immunity
*HEATR6*	Expressed in the nervous system [20]	Growth and Development
*DYNLL2*	Muscle development and muscle fiber growth [21]	Growth and Development
*GRIK2*	Nervous system development [22]	Growth and Development
*HECW2*	Muscle development [23]	Growth and Development
*MDFI*	Muscle development [24]	Growth and Development
*PCNT*	Human dwarfism [25]	Growth and Development
*PRKAB2*	Muscle development [26]	Growth and Development
*RAB19*	Cytoskeletal recombination [27]	Growth and Development
*VEZF1*	Early embryonic development [28]	Growth and Development
*POLR3B*	Human growth and development [29]	Growth and Development
*PCDHA4*	Human neural development [30]	Growth and Development
*ALX1*	The morphology of the beak and the facial development of vertebrates [31,32]	Morphology
*SLC37A3*	Osteoblast [33]	Morphology
*MKRN4P*	Less research	
*TCP11X2*	Less research	

## Data Availability

The original contributions presented in this study are included in the article. Further inquiries can be directed to the corresponding author.

## Abbreviations

The following abbreviations are used in this manuscript:

Fst	Fixation index
KEGG	Kyoto encyclopedia of genes and genomes
NJ	Neighbor-Joining
RADseq	Restriction site-associated DNA sequencing
RJF	Red Junglefowl
π(pi)	Nucleotide diversity
